# Beyond Mortality: Sterility As a Neglected Component of Parasite Virulence

**DOI:** 10.1371/journal.ppat.1005229

**Published:** 2015-12-03

**Authors:** Jessica L. Abbate, Sarah Kada, Sébastien Lion

**Affiliations:** 1 Centre d’Écologie Fonctionnelle et Évolutive (CEFE), CNRS-Université de Montpellier- Université Paul-Valéry Montpellier-EPHE, Montpellier, France; 2 Institute of Ecology and Evolution, University of Bern, Bern, Switzerland; The Fox Chase Cancer Center, UNITED STATES

## Abstract

Virulence is generally defined as the reduction in host fitness following infection by a parasite (see Box 1 for glossary) [1]. In general, parasite exploitation of host resources may reduce host survival (mortality virulence), decrease host fecundity (sterility virulence), or even have sub-lethal effects that disturb the way individuals interact within a community (morbidity) [2,3]. In fact, the virulence of many parasites involves a combination of these various effects (Box 2). In practice, however, virulence is most often defined as disease-induced mortality [1, 4–6]. This is especially true in the theoretical literature, where the evolution of sterility virulence, morbidity, and mixed strategies of host exploitation have received relatively little attention. While the focus on mortality effects has allowed for easy comparison between models and, thus, rapid advancement of the field, we ask whether these theoretical simplifications have led us to inadvertently minimize the evolutionary importance of host sterilization and secondary virulence effects. As explicit theoretical work on morbidity is currently lacking (but see [7]), our aim in this Opinion piece is to discuss what is understood about sterility virulence evolution, its adaptive potential, and the implications for parasites that utilize a combination of host survival and reproductive resources.

**Box 1. Glossary**

**Fitness:** The expected number of adult offspring contributed to the next generation by a focal genotype or phenotype in a given environment.

**Virulence:** Any reduction in host fitness following infection by a parasite. This includes parasite-induced mortality, sterility, and morbidity (other sub-lethal effects of infection).

**Transmission rate:** The rate at which susceptible hosts become infected when encountering infected hosts.

**Basic reproductive ration (*R***_**0**_ **):** The expected number of secondary infections produced by an infected host in an otherwise uninfected population. If *R*_0_ is strictly larger than 1, the disease can spread.

**Parasite fitness:** The expected number of secondary infections produced by a given parasite genotype in a population infected by one or several resident genotypes. As explained in Box 3, there is a simple link between *R*_0_ and parasite fitness only when the epidemiological feedbacks are of a particularly simple kind.

**Epidemiological feedback:** Any environmental variable, such as the density of susceptible or infected hosts, that affects parasite fitness.

**Box 2. Examples of Pleitropic Virulence Components**

Beyond reducing resources allocated to host maintenance and growth (mortality virulence and morbidity), parasite-induced reduction in fecundity (sterility virulence) is a widely-studied and important consequence of host exploitation (reviewed in [13]). Additionally, many parasites have been shown to impact both survival and reproduction of their hosts. For example, commonly sterilizing syphilis and gonorrhea infections in humans [57] caused much mortality and morbidity in the pre-antibiotic era [58–60]. Likewise, the bacterium *Pasteuria ramosa* is considered a “sterilizing pathogen” (e.g., [61]) of the small crustacean *Daphnia magna*, as the host produces parasite spores instead of eggs; nevertheless, *P*. *ramosa* also causes premature death, a process necessary for transmission of the spores [62–65]. Other examples include everything from nematode infections of red grouse [66] to viral, crop-destroying infections of their insect vectors [67] and *Puccinia* spp. fungal infections of European weeds [68]. While for many pathogens sterilization may only be a consequence of host response to infection, some are able to manipulate such responses [69], and specific targeting of diverse host tissues can also occur. For instance, highly prevalent human herpes simplex viruses (HSV-1 and HSV-2) infecting a wide range of neuronal, ocular, mucosal, and epithelial tissues [70] also localize in sperm and spermatozoa, likely affecting male infertility [71] in addition to juvenile and adult morbidity and mortality [72,73]. Similarly, death and morbidity from the human immunodeficiency virus (HIV) infection of blood cells receives a great deal of much-deserved attention, but very little has been done to investigate the potential fertility impacts of elevated sperm abnormalities associated with asymptomatic infection [74].

Diseases in which morbidity effects occur in addition to mortality and/or sterility are far more obvious, if not ubiquitous (e.g, sores, rashes, fever, etc.). Morbidity is often measured in addition to, or as a proxy for, mortality effects, such as in experiments on the evolution of virulence in malaria (e.g., maximum number of red blood cells destroyed [75]), or for "aggressiveness," as it is termed for phytopathogens (e.g., the number or size of tissue lesions, reviewed in [76]).

## Basic Theory

A key quantity in epidemiology is the basic reproductive ratio, *R*_0_, which is defined as the average number of secondary infections produced by an infected host in an otherwise uninfected population [8,9]. In most classical models of horizontally transmitted infections, *R*_0_ is simply a function of parasite transmission and host survival (Box 3). Early evolutionary models emphasized a particularly simple result: selection should favor the parasite strain that maximizes *R*_0_ (Box 3). Consequently, the evolution of virulence is solely constrained by the shape of the trade-off between parasite transmission and host survival [6]. Importantly for our purpose, parasite fitness is then predicted to be independent of host fecundity.

**Box 3. Parasite Fitness, *R*_0_ Maximization, and Epidemiological Feedbacks**

***R***_**0**_ **and parasite fitness**. Classical epidemiological theory shows that parasite persistence in the host population depends on the average number of secondary infections produced by a parasite in an uninfected population [6,9]. This defines the basic reproductive ratio, *R*_0_. In most textbooks, the following expression is found:

$$R_{0}=\frac{\beta }{\mu +\alpha +\gamma }.$$ (1)

Simply put, *R*_0_ is the product of the transmission rate, *β*, and of the average lifespan of infected hosts, 1 / (*μ* + *α* + *γ*), where *μ* is intrinsic mortality, *α* is mortality virulence, and *γ* is the clearance rate.

In fact, for many classical models, there is often a simple link between parasite fitness and *R*_0_ [6,9], and one may write

$$W=\frac{\beta_{m}}{\mu +\alpha_{m}+\gamma_{m}}\hat{S}=R_{0,m}\hat{S},$$ (2)

where the *m* subscript indicates the dependency of the traits on parasite genotype. Eq 2 shows that the fitness of the focal mutant strain, W, is simply the product of the mutant parasite’s basic reproductive ration, *R*_0,*m*_, and of the density, $\hat{S}$, of susceptible hosts in the population at equilibrium. The mutant parasite is then favored by selection if W>1. The dependency on $\hat{S}$ illustrates the role of epidemiological feedbacks: parasite fitness depends not only on how well a parasite strain performs in an uninfected population (which is measured by *R*_0_), but also on how many susceptible hosts it encounters in an already infected population.

***R***_**0**_ **maximization.** Under the simplest ecological scenarios typically assumed, $\hat{S}$ does not depend on host fecundity and is simply given by 1 / *R*_0,*w*_, where *R*_0,*w*_ is the basic reproductive ratio of the wild-type, resident strain. Then, the parasite fitness can be written as a ratio of basic reproductive ratios,

$$W=\frac{R_{0,m}}{R_{0,w}},$$ (3)

and the strain with the larger *R*_0_ is selected for. Evolution will then lead to a gradual increase in *R*_0_, until the maximal *R*_0_ is reached. The precise optimal strategy of the parasite is determined by the trade-off between epidemiological parameters such as transmission, recovery, and virulence [6,9]. For our purpose, an important implication is that host fecundity does not affect the evolutionary outcome, which only depends on parasite transmission and on the survival of infected hosts.

**Epidemiological feedbacks: an example.** It is important to realize that this simple result only holds true under very restrictive epidemiological assumptions (see main text). Consider multiple infections, for instance. If infected hosts can be infected with probability *σ* (superinfection), the fitness of a mutant strain takes the following form [39]:

$$W=\frac{\beta_{m}\;(\hat{S}+\sigma \hat{I})}{\mu +\alpha_{m}+\gamma_{m}+\sigma \beta_{w}\hat{I}}.$$ (4)

Now, parasite fitness depends on the densities of both susceptible and infected hosts. Consequently, host fecundity may feed back on the density of infected hosts and affect the evolutionary outcome [19,39,77].

We argue that this textbook result may have played a role in fastening attention on mortality virulence. Within this framework, sterility virulence can only evolve if there is a trade-off between sterilization and the other life-history traits (transmission and survival) [10]. For instance, there is evidence that sterilized, infectious hosts may sometimes have higher contact rates because of increased longevity or promiscuity [11–13], particularly for sexually transmitted infections [14]. If there is a positive linear relationship between transmission and sterilization, parasites are predicted to fully sterilize their host whenever possible [10,15]—a take-home message that has been largely influential in the literature (e.g., [16]). However, the key finding from these theoretical studies was that other ecological assumptions can favor sub-maximal sterility, and empirical evidence suggests that sterilization need not be perfect [17].

As a result of this focalization on mortality virulence, the potential adaptive nature of sterility virulence for the parasite has often been downplayed (but see [18]), and observed changes in host fecundity following infection have generally been interpreted as resulting from various host responses. For instance, hosts may adjust their resource allocation between fecundity and survival following infection [18–22], or suffer reduced fecundity because of immunopathology [23,24]. Likewise, incomplete host sterility following infection by a “sterilizing pathogen” is usually explored as a result of host resistance mechanisms [25] or imperfect specificity [16], rather than the evolution of a less virulent parasite. We think this is not the full picture. In the remainder of this essay, we stress that epidemiological feedbacks may have non-trivial effects on the evolution of two key aspects of parasite virulence: which host life-history traits are affected (e.g., mortality and/or fecundity) and the magnitude of virulence (e.g., to what extent host survival or fecundity is reduced).

## Implications of Realistic Epidemiological Feedbacks

Importantly, the simple link between *R*_0_ and parasite fitness (explained in Box 3) only holds true under relatively stringent assumptions about the feedback between epidemiological dynamics and selection. Theoretical studies show that realistic epidemiological feedbacks will cause parasite fitness to depend on additional factors that are likely to be affected by host fecundity. Thus, selective effects due to changes in host fecundity cannot be dismissed a priori. Although this has long been recognized for vertically transmitted parasites, the fitness of which necessarily depends on host reproduction [15], the importance of host fecundity for horizontally transmitted parasites seems to have been underplayed.

Here, we highlight four key ecological factors under which host fecundity is predicted to affect parasite fitness.

### Spatial structure

The theory developed in Box 3 considers a well-mixed population where all parasite strains have access to the same amount of susceptible hosts. However, most host–parasite interactions are spatially structured, and this causes different parasite strains to experience different densities of susceptible hosts. As a result, *R*_0_ is no longer maximized by selection because of indirect kin selection effects [26,27]. Kin selection can then favor intermediate levels of sterilization [10,28], because higher fecundity in infected hosts will tend to relax local competition for susceptible hosts [27]. The key point is that, in spatially structured populations, parasites that differ in their allocation between mortality and sterility virulence will tend to have different opportunities for horizontal transmission.

### Epidemiological fluctuations

Many natural host–parasite interactions exhibit fluctuations in host densities, either because of intrinsic nonlinear dynamics or because of external forcing (e.g., seasonality, variation in vector density, etc.). Such non-equilibrium dynamics may, for instance, cause fluctuations in the density of susceptible hosts, thereby affecting short- and long-term selective pressures on parasite traits [29–34]. In general, one may expect the non-equilibrium density of susceptible hosts to be affected by the fecundity of infected individuals. By taking this into account, novel theoretical approaches building on quantitative genetics methods [30,32] may help shed light on the origin and maintenance of sterilizing parasites.

### Multiple infections

Most natural infections consist of several parasite strains or species [35–37]. In hosts infected by multiple parasite strains, the fitness of a focal strain will be reduced by both host mortality and by the rate at which susceptible hosts become infected by other genotypes (see Box 3). Parasite fitness then depends on the density of infected hosts, and this creates a new route by which an effect of parasites on host fecundity may feed back on parasite evolution [38–40]. For instance, the level of sterilization in the population will affect the selective pressures on a mutant parasite with a different virulence strategy.

### Variation in host quality

While classical theory considers that all hosts have the same value for the parasite, natural host–parasite interactions often exhibit variation in host quality (e.g., because of natural resistance, treatments, or other forms of class structure such as sex-based differences in immunity). The parasite is then faced with the problem of exploiting a heterogeneous resource. As a result, the optimal strategy for the parasite is determined by the frequencies of each type of host [41–43]. In general, these frequencies may be expected to be affected by host fecundity.

These four ecological factors are ubiquitous in natural host–parasite populations and, importantly, are often combined. Thus, under realistic ecological scenarios, we should not expect differences in host fecundity to be irrelevant to the evolution of horizontally transmitted parasites. Epidemiological feedbacks alter the classical predictions of Box 3 and create novel selective pressures that may affect how a parasite should exploit the resources the host allocates to survival and fecundity. So far, we only have a partial theoretical picture (mostly limited to the effect of spatial structure [10]). Combined with genetic constraints (e.g., trade-offs between transmission, sterility, and mortality virulence), epidemiological feedbacks potentially introduce a whole range of possible evolutionary outcomes. In addition, the evolution of parasite strategies of host exploitation may feed back on population dynamics, potentially affecting host and parasite extinction rates.

## From Theoretical to Empirical Studies

We think the focus on classical epidemiological models has contributed to neglecting the potential of sterility virulence as an adaptive trait in the theoretical literature. Taking into account epidemiological feedbacks suggests that the joint evolutionary dynamics of mortality and sterility virulence may be more complex than previously imagined. Although a number of models have investigated the joint effects of mortality and sterility on the population and coevolutionary dynamics of host–parasite interactions (e.g., [25,44–47]), very few have addressed the evolution of parasite allocation between mortality and sterility virulence. For instance, Thrall, et al. [48] have investigated the evolution of diseases with both sexual and non-sexual transmission routes under the assumption that allocation to sexual transmission leads to reduced fecundity and lower mortality, while van Baalen [49] has studied the allocation between mortality and sterility virulence in a semi-spatial model in which the degree of local (parent-to-offspring) and global transmission may vary. Both results show that the allocation between killing or sterilizing a host is very sensitive to epidemiological feedbacks. Given the abundance, diversity, and sometimes long-standing evolutionary history of systems in which sterility virulence occurs [50], the lack of studies on the topic is striking.

We therefore urge that a general theoretical framework for virulence evolution should take into account the multiple effects of parasite exploitation on host traits. Such a generalized framework would have important implications for empirical studies, as there are some potential pitfalls with the current idea that parasite-induced changes in host fecundity do not affect parasite fitness unless there is an obvious trade-off between transmission and fecundity (e.g., for the sexually transmitted and obligately sterilizing anther-smut fungi [51]). First, if one measures only mortality effects of a given disease, other (minor or less obvious) virulence components may go completely unnoticed. Cowpox virus had long been thought to have no apparent “disease effect” in its wild rodent reservoir until the existence of a negative impact on their fecundity was revealed [52]. In contrast, virulence effects in the well-studied Drosophila–nematode system are systematically measured in terms of both fertility and longevity [53]. Although secondary effects may not always be important or adaptive (e.g., *Ophreocystis electroskirra* effects on Monarch butterfly mating success do not necessarily reduce reproductive output [54]), such practice offers a more thorough understanding of the disease’s natural history. Second, tests of theory-derived hypotheses may be biased if the theoretical framework used does not effectively capture the biology of the system. Third, when thorough measurements of lifetime reproductive success are technically impossible, experimental studies often rely on indirect fitness proxies (e.g., [55]). However, the validity of these proxies is not always established [56]. We argue that mathematical models exploring the adaptive potential of complex host exploitation strategies could help identify which traits need to be measured to calculate a meaningful fitness proxy, and could open avenues of new research into the evolution of virulence in both novel and established host–pathogen systems.

## Conclusion

Although theoretical studies are largely biased toward mortality virulence, extensions of classical theoretical evolutionary epidemiology reveal that host fecundity may very likely matter for parasite evolution, and that parasite strategies that manipulate both the fecundity and survival of the host may be shaped by natural selection under realistic ecological scenarios. Given the widespread occurrence of parasites that, in contrast to earlier predictions, do not fully sterilize their host, we urge theoreticians and empiricists to work together toward a better understanding of the adaptive and multifaceted nature of parasite virulence.
